# An Automatic on Top Analysis of Single Scan Tracks to Evaluate the Laser Powder Bed Fusion Building Parameters

**DOI:** 10.3390/ma14185171

**Published:** 2021-09-09

**Authors:** Alessandra Martucci, Fabrizio Marinucci, Antonio Sivo, Alberta Aversa, Diego Manfredi, Federica Bondioli, Paolo Fino, Mariangela Lombardi

**Affiliations:** 1 Department of Applied Science and Technology, Politecnico di Torino, Corso Duca degli Abruzzi 24, 10129 Turin, Italy; fabrizio.marinucci@polito.it (F.M.); antonio.sivo@polito.it (A.S.); alberta.aversa@polito.it (A.A.); diego.manfredi@polito.it (D.M.); federica.bondioli@polito.it (F.B.); paolo.fino@polito.it (P.F.); mariangela.lombardi@polito.it (M.L.); 2 Center for Sustainable Future Technologies CSFT@Polito, Istituto Italiano di Tecnologia, Via Livorno 60, 10144 Turin, Italy

**Keywords:** laser powder bed fusion, single scan tracks, computer-aided method, additive manufacturing, automatic analysis

## Abstract

The production of dense samples produced by laser powder bed fusion (LPBF) is mainly determined by the choice of the best combination of construction parameters. Parameter optimization is the first step in the definition of an LPBF process for new alloys or systems. With this goal, much research uses the single scan track (SST) approach for a preliminary parameter screening. This study investigates the definition of a computer-aided method by using an automatic on top analysis for the characterization of SSTs, with the aim of finding ranges of laser power and scan speed values for massive production. An innovative algorithm was implemented to discard non-continuous scans and to measure the SSTs quality using three regularity indexes. Only open source software were used to fine tune this approach. The obtained results on Al4Cu and AlSi10Mg realized with two different commercial systems suggest that it is possible to use this method to easily narrow the process parameter window that allows the production of dense samples.

## 1. Introduction

Additive manufacturing (AM) techniques are attracting widespread interest in many industrial fields due to several advantages. In particular, the possibility to produce complex shaped components with a significant freedom of design that can lead to the production of lightweight parts plays an important role in the industrial development of AM techniques [1,2]. Moreover, thanks to the net-shape process further assembling or post-processing joining can be avoided.

In recent years, AM research has focused on laser powder bed fusion (LPBF) technology for metals. This process is based on building metallic components layer by layer using a laser beam to melt specific regions of the powder bed [3]. The quality of the final LPBF part depends on the quality of each single layer and on the interaction between successive layers. The part quality can be controlled trough the building parameters such as layer thickness (t), laser power (P), scan speed (v), hatching distance (hd), and scanning strategy [4]. The most suitable set of parameters to process a powder represents the optimal parameters window. Every time a new alloy is studied or composition changes in known alloys are applied, the optimization of process parameters window is necessary to produce bulk samples without cracks and fusion defects, hence with full density.

The classical approach to find the optimal process parameters window consisted of building small bulk samples, in general cubes, varying the combination of process parameters, and evaluating their final relative density and the presence of pores and cracks. However, building and analyzing one cube for each parameter set is overly time-consuming and low-performing. In order to improve efficiency, the design of experiments (DOE) approach was introduced. DOE is a powerful data collection and analysis tool that deals with planning, conducting, and analyzing controlled tests to investigate parameter sets. Associated with the classical method, the DOE tool accelerated the definition of the parameter window, reducing the number of samples to analyze [5,6].

Recently, for a preliminary process parameter screening, a simpler and faster approach has been set up by exploiting Single Scan Tracks (SSTs) [7,8,9,10,11,12,13,14,15,16]. The SST corresponds to a laser track scanned on a single powder layer previously spread onto a substrate. In contrast to bulk samples, in an SST job, only P and v can be varied, whereas the layer thickness is fixed. The analysis of SST quality can then be correlated with Linear Energy Density (LED), expressed as power over scan speed. The SST approach reduces the time cost and the waste of powder of the first screening, compared to massive samples building, thanks to the decrease in both production and characterization times and the quantity of used powders [14].

In literature SST analyses were performed by analyzing both their cross-section and on top morphology. The most used method is the cross-section analysis, which consists of a complex procedure. According to this method, it is necessary to cut the platform where SSTs were printed, polish the surface and observe the different SST sections using an optical microscope. On the other hand, in on top analysis the SSTs preparation procedure is considerably streamlined. In fact, generally, the as obtained SSTs are directly observed exclusively using an optical microscope [7].

The quality of SST cross-sections is linked to their morphology. According to Aversa et al. and Yadroitsev et al., the analysis of the geometrical features of an SST cross-section, such as its width, growth, depth and contact angles, can be successfully used to investigate the melting and consolidation behavior of the material and thus its AM processability [7,17]. Furthermore, in many works, the correlation between LPBF parameters and the cross-section morphology was considered to identify the process window [7,8,9,17]. As reported in Aversa et al., several considerations on cross-section can be made, such as the fact that stable SSTs are usually characterized by similar growth and depth values although low depth or contact angle values can be correlated with incorrect process parameter values [7]. It is worth stating that a specific correlation between cross-section feature and the optimal process parameters has not yet been defined in the literature.

Additionally, the on top analysis has been used in the majority of investigations to evaluate the effect of the chosen process parameters on the regularity of the SST borders [7,10,11,12,13,14,15,16]. For instance, Childs et al. tried to link the SSTs shape with LED values [10]. In line with this, Wei et al. associated a lower scan speed with a reduced quality of SSTs in terms of their border roughness due to a melt pool superheating which causes evaporation and mass loss of metal material [11]. On the other hand, a higher scan speed seems to produce insufficient energy to completely melt the powders, causing the appearance of a balling effect [9]. To the best of our knowledge, Aversa et al. and Nayak et al. are among the few studies that classified the SST on top morphologies into regular and irregular [7,12]. In particular, following Aversa et al., the on top analysis made it possible to distinguish five kinds of SST shapes related to “insufficient melting” when the LED is too low to melt the powder and no scan track is formed; “balling” when the LED is low and droplets of molten powder are formed; “thin and stable” when the LED is correctly set and the SSTs are continuous and regular; “irregular” when the LED is high and the SSTs are continuous but strongly asymmetrical and “too thick” when the LED is too high and the keyhole melting occurs [7]. Only the “thin and stable” category is linked to the optimal process parameters, whereas the others are considered irregular SSTs.

Furthermore, in the work of Nie et al., the preliminary process parameters P, v and t were defined with an SST job and multi-tracks were produced and analyzed for the definition of hd [13]. Based on Nie’s work, Bosio et al. demonstrated how suitable hd values can be also defined through the evaluation of the SST width and the desired overlapping between two consecutive laser tracks [14]. In Bosio et al.’ work, bulk samples were produced varying the hd values based on different overlapping and then characterized. Their results proved that an overlapping in the range of 0–20% results in a higher density level [14]. This update makes the SST approach more useful in terms of time saving and cost-effectiveness.

Despite the interest shown in SST on top analysis, the above studies merely used this analysis to exclude SSTs with visible defects. In fact, they usually arrive at the definition of bulk process parameters with the help of other techniques such as cross-section analysis. Furthermore, the on top analysis carried out so far strongly depends on the sensitivity of the operator that analyzes the images and it is therefore heavily subjective. 

The purpose of this study is to expand the on top analysis giving an automatic computer-aided method to evaluate SST characteristics. With the goal of ensuring an even more rapid and accurate analysis, the proposed on top method provides the use of a software that automatizes the whole process, minimizing the operator contributions and the time required. The algorithm first performs an automatic selection of the continuous SSTs, discharging the defective ones, and then it measures their quality using three proposed regularity indexes. The algorithm is reported in the Supplementary section.

## 2. Materials and Methods

The method was validated using two different aluminum alloys (AlSi10Mg and Al powder containing 4.5 wt% Cu) and two LPBF systems, one lab scale and the other industrial scale. Laser power, scan speed and layer thickness were varied in order to evaluate their effect on the SSTs regularity by the automatic on top analysis.

### 2.1. AlSi10Mg Alloy

A commercial gas atomized AlSi10Mg powder, supplied by Concept Laser (commercial name CL31), was selected for this study and used after sieving below 40 μm. The CL31 composition is shown in Table 1.

A Concept Laser Mlab cusing R was used to process the AlSi10Mg powder. This system is a lab scale system with a 9 × 9 cm^2^ platform equipped with a fiber laser having 100 W as maximum power, a 1070 nm as wavelength and a laser spot of 50 μm.

The powder was mixed with ethanol 50 vol% in order to facilitate the layer deposition. For each job, a single powder layer was accurately spread on a commercial pure aluminum platform using a self-developed recoating system. The platform was then heated up to 100 °C with an external system in order to evaporate all the solvent.

AlSi10Mg SSTs were produced varying P in the range 75–95 W and v in the range 350–950 mm/s with a layer thickness of 20 or 25 μm.

### 2.2. Al4Cu Alloy

To evaluate the robustness of the method, an Al alloy gas atomized powder containing 4.5 wt% Cu (named in the following Al4Cu) supplied by the Universität Bremen was also tested using an EOSINT M270 Dual mode system. This powder was sieved below 50 μm.

The EOS system is an industrial scale system with a 25 × 25 cm^2^ platform equipped with Yb-fiber laser with a nominal maximum power of 200 W, a wavelength of 1070 nm and a spot of 100 μm.

The powder was mixed with ethanol 50 vol% in order to facilitate the layer deposition. A single powder layer was accurately spread on a pure aluminum disc placed in a modified platform using a self-developed recoating system [7]. Before starting to scan the SSTs, the building platform was preheated at 100 °C for 1 h.

Al4Cu SSTs were built varying P in the range 100–195 W and v in the range 300–1500 mm/s with a layer thickness of 50 μm.

### 2.3. Image Acquisition

At first, each single scan track was characterized on top through the use of a LEICA DMI 5000 M optical microscope. For each SST, nine 100× images were acquired and numbered 1–9, in order to cover the entire SST length. In order to limit the edge effect of the scan process and to be sure to analyze a section where the steady state was reached, the script selected only the images 3–7, thus excluding the two first and last images. Taking these frames, the main focus was to capture the SST as central as possible in the screen to facilitate the image processing. For each image microscope parameters, i.e., aperture, field and illumination, must be maintained equal to repeat the same conditions.

**Figure 1:**
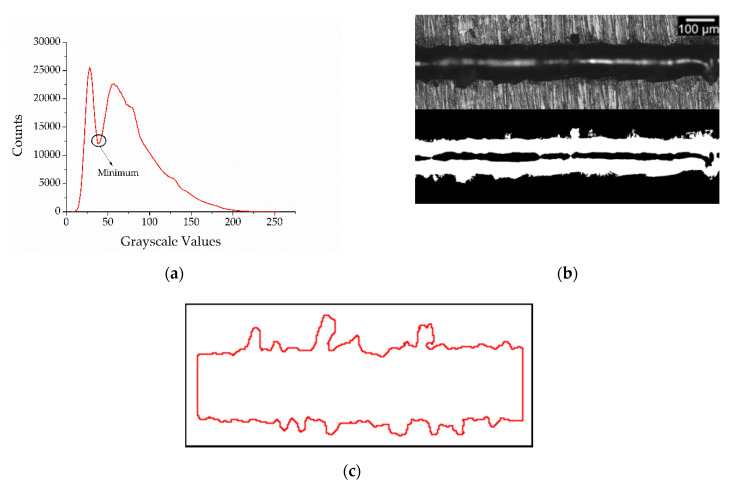
Example of the greyscale histogram used to realize the automatic thresholding (**a**), the original image transformed in a binary one (**b**) and ROI elaborated by the software (**c**).

After these operations, the images were elaborated for the subsequent quantitative analysis. In the first step, a threshold was applied to convert the greyscale image to a binary image. To optimize the automatic threshold, the proposed script used the grey scale histogram that the Leica software automatically generated for each image. In particular, to correctly discern an SST from the platform, the script used the minimum of the curve (Figure 1a). Each image was then converted to a binary image (Figure 1b). In order to automatically evaluate the single scan track continuity, the definition of SST profiles was needed. For this purpose, the binary images allow the SST profiles to be easily distinguished from the substrate and then region of interest (ROI) was defined for each image (Figure 1c).

**Figure 2:**
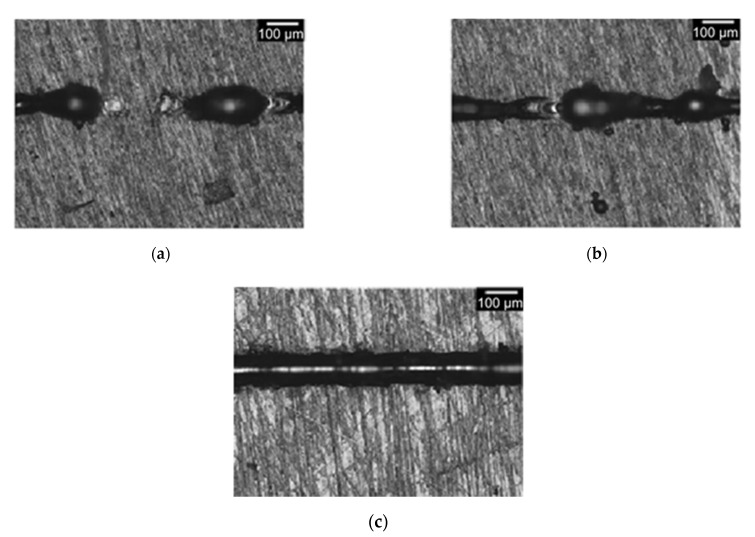
Optical Micrographs of “irregular” SSTs (**a**–**b**) and a “regular” SST (**c**).

In line with what was reported by Aversa et al. and by Nayak et al., varying laser power and scan speed different morphologies can be observed [7,12]. The implemented software is able to count SSTs closed profiles. A discontinuity is then identified by the software for each image with more than one closed profile. In this way, discontinuities can be detected in SSTs with important melt failures (Figure 2a) and ones with small substrate melts (Figure 2b). When there are more than two images with discontinuities in the entire SST, the software identifies SST as “irregular”. On the other hand, SSTs with only one closed profile (Figure 2c) are classify as “regular” and then selected for the analysis.

**Figure 3:**
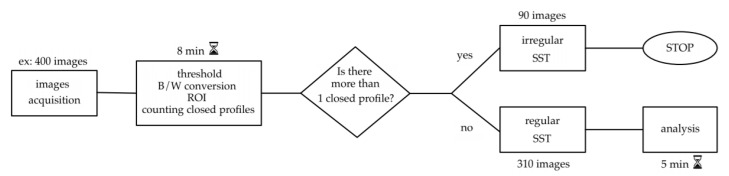
Flowchart outlining the method steps from image acquisition to final analysis with an example of processing time.

The selection approach of the implemented software is schematized in Figure 3, where the processing time needed for classify 400 images is also reported.

As reported in Figure 3, the processing time needed to discern 400 images is about 13 min (8 min for the pre-processing and 5 min for the final analysis with the definition of the three regularity indexes).

### 2.4. Regularity Indexes 

The quality of the SSTs was assessed by the proposed software with three different regularity indexes: 

1. Perimeter index

2. Roughness index

3. Width standard deviation (STD) index

**Figure 4:**
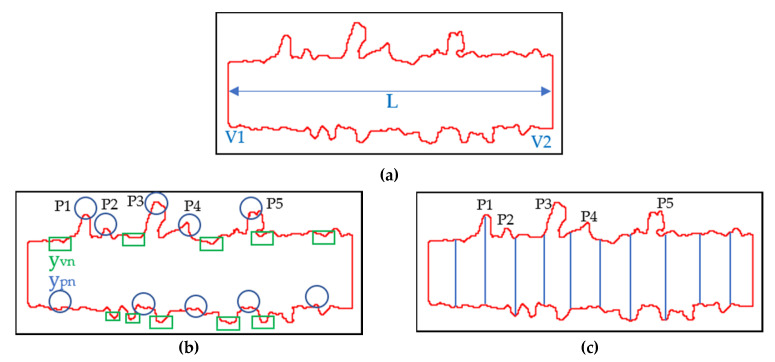
Examples of: the ROI perimeter index (**a**); the ROI roughness index (**b**); the ROI width STD index (**c**).

The first regularity index, related to the scan perimeter, was obtained by the Equation (1):

(1) $$\mathrm{index}\;1=P_{\mathrm{real}}-P_{\mathrm{ideal}}$$

where P_real_ is the real ROI perimeter, calculated by software and P_ideal_ is obtained as the sum of V1, V2 and 2L according to the scheme reported in Figure 4a. The vertical segments are calculated as the difference between the y-values of the upper and lower borders considering their first points (V1) and their last points (V2). Moreover, the ROI length, referred to as L, is calculated as the distance between the x-values of the midpoints of V1 and V2. It is clear that a smaller index value in pixel implies a greater SST regularity. Conversely, a high value of this index could indicate SSTs affected by balling.

The second proposed index evaluated the profile irregularity by the roughness measurement, Rz, as the mean distance in pixel among the five highest peaks (in Figure 4b in blue) and five lower valleys (in Figure 4b in green), identified on the of the upper and lower borders of the SST using the Equation (2):

(2) $$\mathrm{index}2=R_{z}=\frac{\left(y_{p1}+\ldots +y_{p5}\right)-\left(y_{v1}+\ldots +y_{v5}\right)}{5}$$

where $y_{\mathrm{pn}}\;$stands for the y-value of the n-peak and${\;y}_{\mathrm{vn}}\;$for the y-value of the n-valley. Index 2 gives local information to the linearity of the lower and upper borders of the SST. As in index 1, the lower the index value in pixel, the greater the linearity of the profile. On the contrary, a high value of this index means that SSTs are strongly asymmetrical and irregular.

The software evaluates the index 1 and index 2 values for the images 3–7 and then the average value is calculated.

The last index exploited the SST width measures (Figure 4c), and in particular it is based on the determination of the STD of repeated measurements. The width values are obtained as the difference between y-values of 10 equally spaced points on the upper and lower borders. Then, the index 3 can be calculated using the following Equation:

(3) $$\mathrm{index}\;3={\;\sigma }_{\mathrm{tot}}=\sqrt{\overset{n}{\underset{i=1}{\sum }}\sigma_{i}{}^{2}}$$

where $\sigma_{i}\;$is the STD of the width measured on a single image and $\sigma_{\mathrm{tot}}\;$is the STD of the SST width measured using five images. 

This index gives results about the global SST regularity. It follows that the lower the index 3 value in pixel, the higher global SST regularity due to a lower variance of the width. 

It is important to note that the index 2 and index 3 are very similar but at the same time complementary. In fact, as observed in Figure 4b,c, the information related to the Peak 1 (P1) is well detected by both indexes, but index 3 lost the information related to Peaks 2, 3, 4 and 5 (P2, P3, P4 and P5). This is due to the nature of index 3, based on sampling measurements, so affected by a statistical error. This suggests that the use of several indexes is useful and makes this approach more robust, avoiding loss of information.

In this step, the script could also calculate the mean width of SST in order to have information about hd right value, as defined by Bosio et al. [14].

**Figure 5:**
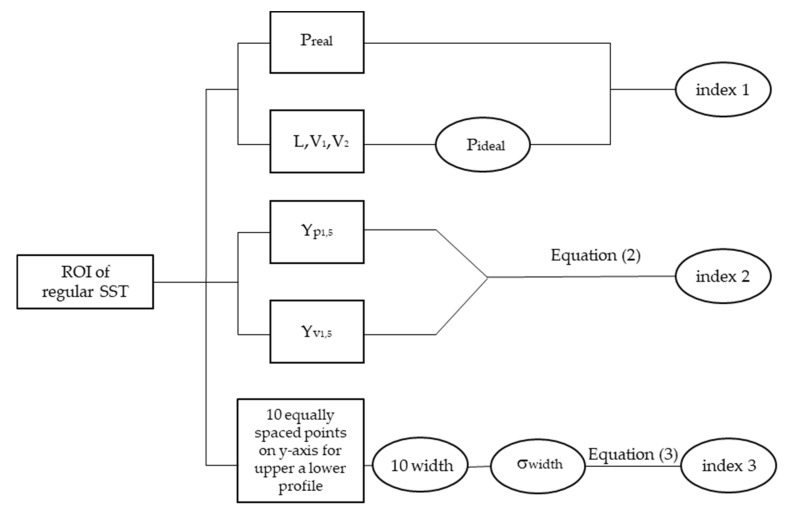
Method steps of the implemented software for the definition of the regularity indexes.

The software works evaluating step by step all indexes for each image following the flowchart of Figure 5 and returning the final value of regularity indexes in a .csv file that is automatically created. 

## 3. Results

In this paragraph, the results of the proposed methodology for the automatic investigation of SSTs quality were reported.

### 3.1. AlSi10Mg Alloy

**Figure 6:**
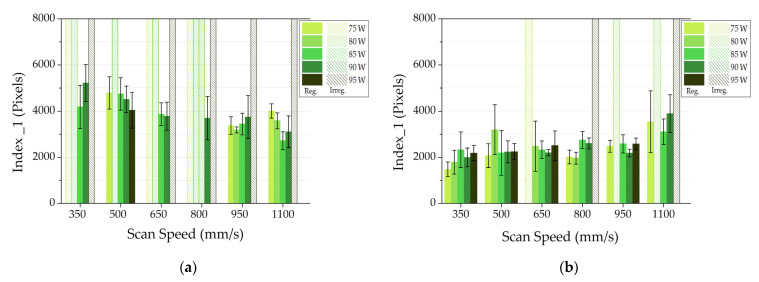
Index 1 for AlSi10Mg SSTs built with a layer thickness of 20 μm (**a**) or 25 μm (**b**).

Figure 6 reports the index 1 values for AlSi10Mg SSTs and their STD. In these graphs, in order to give a complete overview of the analysis, the authors decided to also show power and scan speed conditions corresponding to the discontinuous SSTs that the software discarded in the first phase of the analysis. The irregular SST are represented using full scale bars with patterns in order to highlight them.

Observing the full scale bars with patterns in Figure 6a, it is evident that a significant number of AlSi10Mg SSTs built with 20 μm layer thickness were discarded due to the presence of discontinuities. On the other hand, in Figure 6b, related to SSTs made of AlSi10Mg with 25 μm layer thickness, it is observable that all SSTs were almost accepted. In this graph, the values of index 1 were globally lower than those obtained with 20 μm as layer thickness, confirming a higher SST regularity. In particular, observing Figure 6b, the higher values of index 1 for SSTs realized with scan speed of 1100 mm/s and the higher standard deviations for SSTs scanned with scan speeds of 350 and 500 mm/s suggest that these parameters are not suitable for the construction of bulk samples.

**Figure 7:**
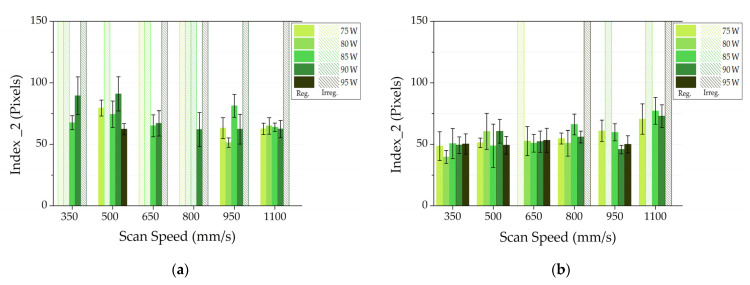
Index 2 for AlSi10Mg jobs built with a layer thickness of 20 μm (**a**) or 25 μm (**b**).

Figure 7 reports the values of index 2, related to the SSTs roughness, for both layer thicknesses. It can be noticed that the behavior of index 2 is similar to that of the index 1 and the results are comparable. In fact, lower index 2 values are detected with 25 μm as layer thickness, so these SSTs seem more regular. Analyzing Figure 7b, the higher values of the index 2 were obtained for SSTs realized with 1100 mm/s as scan speed and the higher standard deviations were recorded for SSTs scanned with a scan speed of 500 mm/s. Therefore, all conditions with these scan speed values cannot be considered for the production of bulk samples.

**Figure 8:**
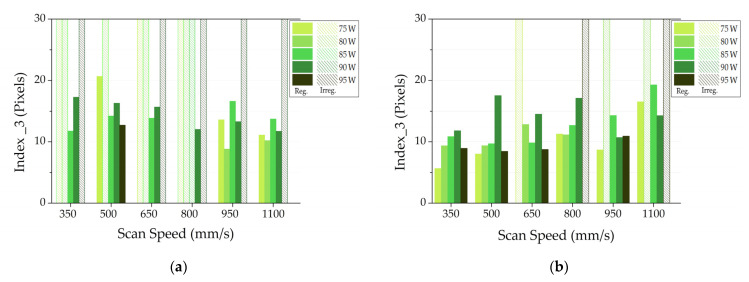
Index 3 for AlSi10Mg jobs built with a layer thickness of 20 μm (**a**) or 25 μm (**b**).

Finally, Figure 8 illustrates the results related to index 3. Additionally, in this case, the job with a layer thickness of 20 μm exhibits an overall higher index 3 value. Figure 8b reports the higher index values for the SSTs realized with 1100 mm/s as scan speed. Moreover, the too high index 3 values suggest to also discard 90 W as power value.

### 3.2. Al4Cu Alloy

**Figure 9:**
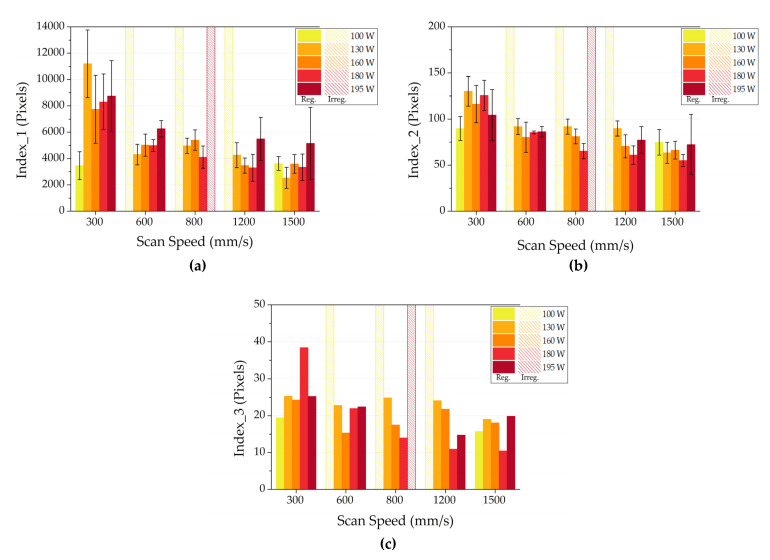
Index 1 (**a**), Index 2 (**b**) and Index 3 (**c**) for Al4Cu alloy.

To evaluate the robustness of the proposed method, the analysis was performed also on Al4Cu SSTs obtained with a different LPBF system. Additionally, in this case, the method can provide important information to obtain a narrow process parameter window. In Figure 9, the behavior of the three regularity indexes is reported as a function of scan speed and laser power. In this case, it is easily visible that all the indexes decrease as the scan speed is increased. This behavior suggested that, in the analyzed range, the tracks are more regular and characterized by less roughness as the scan speed increases. The homogeneity of the tracks can be appreciated by the analysis of both the index 3 and the standard deviations of index 1 and 2 values. 

## 4. Discussion

Observing the full scale bars with patterns referred to AlSi10Mg SSTs with 20 μm as layer thickness in Figure 6a, Figure 7a and Figure 8a, it is possible to state that this layer thickness can be discarded for the subsequent production of bulk samples. Moreover, paying attention to the index values and their standard deviations of AlSi10Mg SSTs produced with a layer thickness of 25 μm (Figure 6b, Figure 7b and Figure 8b), it is easy to define a narrow process parameter window that ensures more regular SSTs. In particular, a layer thickness of 25 µm, powers of 80 and 85 W and scan speed from 650 to 950 mm/s will be considered for the production of bulk samples.

**Figure 10:**
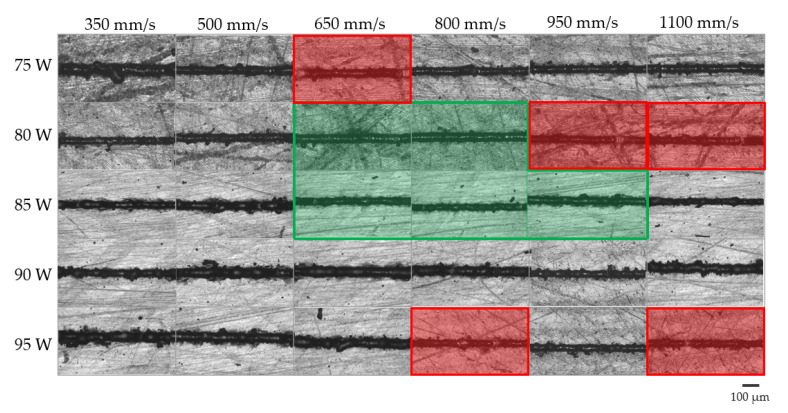
Process window with optical micrographs of on top AlSi10Mg SSTs with a layer thickness of 25 µm.

In order to validate the results of the implemented software analysis, in Figure 10, the micrographs of on top morphologies of AlSi10Mg with a layer thickness of 25 µm are reported. In some conditions highlighted in red in Figure 10, discontinuities are easily visible. These SSTs correspond to the full scale bars with patterns in Figure 6b, Figure 7b and Figure 8b because the software classified them as irregular. On the other hand, when SSTs are continuous, the software analyzes them for calculating the three index values. On the basis of the analysis results, only few process parameters are considered for the bulk production. These power and scan speed sets correspond to the continuous and more regular SSTs of Figure 10 highlighted in green.

According to the script the SST produced with P = 85 W and v = 650 mm/s presents a mean width value of 70.8 µm. Consequently, as suggested by Bosio et al., an hd of 70 µm should be fixed for guaranteeing an overlapping of about 0% [14].

**Figure 11:**
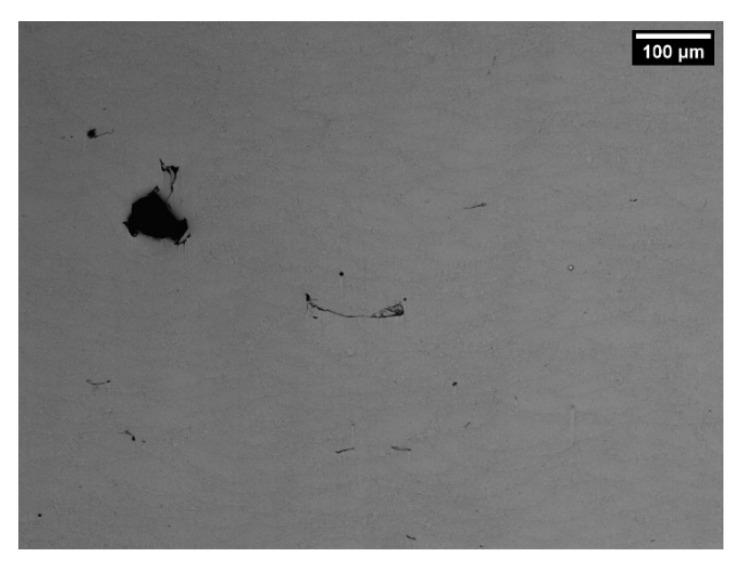
Cross-section morphology of sample built with P = 85 W, v = 650 mm/s and hd = 70 µm.

In order to verify the relationship between the discovered process parameters and the optimal densification, bulk samples were produced with P = 85 W v = 650 mm/s hd = 70 µm and with island scanning strategy. In Figure 11, an example of the cross-section morphology of these bulk samples is reported. A relative porosity level of 1.2% with a standard deviation of 0.4% (on 10 measurements) is reached. This demonstrates how the process parameters suggested by the implemented software are useful to achieve optimal densification levels on bulk samples. Further studies on the scanning strategy can be carried out to additional improvements in densification.

In the case of Al4Cu powder with a layer thickness of 50 µm, powers between 130 and 180 W and scan speed between 600 and 1200 mm/s will be considered to produce bulk samples. According to the script, when scan speed is 600 mm/s a mean width value of 137 µm is recorded, whereas for a speed of 1200 mm/s a mean width of 1100.0 µm is measured. Consequently, as suggested by Bosio et al., in order to guarantee an overlapping of about 0%, an hd of 137 µm should be fixed for a scan speed of 600 mm/s and an hd of 110 µm for 1200 mm/s as scan speed [14].

## 5. Conclusions

LPBF production requires the definition of the optimal process parameters window through an analysis of SSTs or massive samples.

Manual inspection of large numbers of SST samples is time consuming and subject to operator experience, resulting in variable and erroneous results. Computer-aided analysis with open source software overcomes this problem and provides more accurate methods for a quantitative image analysis for the evaluation of SSTs regularity. According to this method, it is possible to obtain a first screening of the process parameters (power, scan speed, layer thickness and hatching distance) with which massive samples can be produced.

The method proposed in this work is able to analyze a wide range of process parameters providing, within minutes, important information to narrow the process parameter window. The analysis is automatic and based on three different regularity indexes to define the regularity and homogeneity of the SSTs.

The main results can be summarized as follows:

- On top images give information about SST quality analysis, allowing the classification in irregular or regular SSTs;
- The proposed method allows an impartial comparison of regular SSTs through a quick image analysis and the index definition;
- The proposed method allows a first screening of the process parameters (power, scan speed, layer thickness and hatching distance) to be carried out in only a few minutes;
- The proposed method is validated for different materials or production systems.

## Figures and Tables

**Table 1:** AlSi10Mg chemical composition.

Element (%)	Si	Fe	Cu	Mn	Mg	Ti	Al
AlSi10Mg	9–11	≤0.55	≤0.05	≤0.45	≤0.2–0.45	≤0.15	Remainder

## Data Availability

The data presented in this study are available in supplementary material.

## Supplementary Materials

The following are available online at https://www.mdpi.com/article/10.3390/ma14185171/s1, Script 1.jim (threshold and extracting ROI script), Script 2.jim (ROI analysis script).
