# Supplementary material for: An Automatic on Top Analysis of Single Scan Tracks to Evaluate the Laser Powder Bed Fusion Building Parameters
Source: Materials (Basel). 2021 Sep 9;14(18):5171. doi: 10.3390/ma14185171 (PMC8472349; doi:10.3390/ma14185171)
Supplement: Supplementary file 1 [file materials-14-05171-s001.zip › Script 1.pdf]

```
/*
Script 1: Finding ROIs (the board of SST) and discarging defective zones.

INPUT: Images Folder, obtained by optical microscope, following the coditions decribed in section
"Materials and Methods"

OUTPUT:
-"Discarded_Images" folder (showing masks)
-"ROIs" Folder, where ROIs are saved as files ".roi"
*/

//String to int Function (Used to place "Estimate" table)
function f2i(f) {
	i = parseInt(d2s(f, 0));
	return i;
}

// Start Variables Definition //

extension= ".tif";  //Image Extension definition: the user can choose the extension of the images in input

xth = 20;	//Image Width cutoff (portion of ROIs discarded near edges)

crop_offset = 114;  //Crop offset: chosen according to SST centering

low_area_lim=40000; //Area limit to exclude noise [pixels]

// Saves screen dimensions [pixels]
sw = screenWidth;
sh = screenHeight;

//Flags
```

```
con=0;                                                            //ROIs control
Condition

lag=0;                                                            //Ending Analysis
Condition



//"Estimate" Table, where are saved the analyzed Images labels

row =-1;                                                          //Starting row

table1 = "Estimate";                                     // Creating table and title

Table.create(table1);


//Table dimensions

width=400;

height=300;


//Table Position

Table.setLocationAndSize(f2i(sw/4),f2i(sh/2), width, height);


run("Set Measurements...", "area fit display redirect=None decimal=2"); //Measurment settings


run("Clear Results");                              //Clear "Results" table, in order to have
starting condition

setBatchMode(true);                              //Starting Batch Mode: this mode can hide
the analysis on going showing only the final result


TIME1=getTime();                   //Time variable of starting analysis (Used for time of calculation-TOC)


//End Variables Definition//

/*____________________________________________________________________________________*/


//Folders manager

//Input/output Folders management

dir1 = getDirectory("Choose Source Directory "); //User chooses the starting folder (Images folder)
```

```
dir2= dir1 + "Discarded_Images\\";            //"Discarded_images" folder

dir3=dir1 + "ROIs\\";                         //"ROIs" folder

File.makeDirectory(dir3);                     //Folders Creation

File.makeDirectory(dir2);



// Reading files in input Folder

list = getFileList(dir1);                     //Saves Images Labels in "list" Array

for (i=0; i<list.length; i++) {

if (endsWith(list[i], "/"))                   //End analysis condition: If a generic "list" is not an image, is saved with "/" at the end. When the folder is out of images to read, analysis stops.

        lag=1;                                                          //Updates exit flag

    else if (endsWith(list[i], extension))

        processImage(dir1, list[i]);      //Applies "Processing image funcion" to each image

}

/*____________________________________________________________________________*/


//Processing image function

function processImage(dir1, name) {

row++;                                                                  //Update "Estimate" Table Rows number

Table.set("Label", row, name);                //Setting Column name and current image label

open(dir1+name);                              //Opening the image to process


// Extracting Image name and cut the file extension from it

dotIndex = indexOf(name, ".");

fname = substring(name, 0, dotIndex);


// Exctracting the less character of image name which is the number of the image acquired

end=lengthOf(fname);

ln=substring(fname, end-1, end);

imag_no=parseInt(ln);
```

```
if((imag_no<1) || (imag_no>10)){              //Condition necessary to discard the edge of SST, near the contour, so to analyze only the central portion

            close();

            }else{

setBatchMode(false);                       //Allows to see the process on going, if is set "True" the script will work in background


//Setting scale in order to measure in pixel or other Unit of measure

run("Set Scale...", "distance=1 known=1 pixel=1 unit=um");

getPixelSize (unit, pixelWidth, pixelHeight);





            // Cropping image

            x = sw/2;

   y = sh/2;

            makeRectangle(0, 153, 2048, 1200);

            run("Crop");

            run("8-bit");

            selectWindow(name);

            run("Set... ", "zoom=33.3");

            w = 2048;

            h = 1000-crop_offset;

            ar = f2i(w/h);

            q = 1.8;

            setLocation(0,0,f2i(sw/q),f2i(sw/(ar*q)));


// Exit condition of loop

param = 1;


            do{     //Starting image Analysis
```

```
//Duplicating image and getting title

selectWindow(name);

run("Duplicate...", " ");

name1 = getTitle();

/*_______________________________________________________________________________________*/

//Start Auomatic Threshold Identifier: Find the minimum between two Maxima in the typical GreyScale Histogram (descibed in "Materials and Method"), in order to have the best value of Threshold


//Convolution counter definition

deg=0;


//Getting and saving histogram from the image

getStatistics(area, mean, min, max, std, histogram);

conv=Array.copy(histogram);             //Array which contains the histogram values after convolution


for (i = 120; i < histogram.length; i++) { //Correcting histogram Cycle where are excluded the last 200 values of histogram, considered as noise

        histogram[i]=0;

}




Array.getStatistics(histogram, min, max, mean, stdDev);  //Finding the absolute max value of histogram and saving his position in "pos"

for (i = 0; i < histogram.length ; i++) {

        if(histogram[i]==max){

                pos1=i;

                }

        }



//Convolution: three point convolution on histogram, repetead 10 times (deg=10)
```

```
while(deg<=10){                                                                          //Starting
Convolution: this step is necessary to smooth histogram function and so to find easily the ideal threshold
value

j=0; //Counter

for (i = 2; i < histogram.length-1; i++) {

        conv[j]= (conv[i-1]+conv[i]+conv[i+1])/3;

        j++;

        }

deg++;

}


//Cutting convoluted Histogram to exclude noise

conv1=Array.copy(conv);                          //New Convolution array definition

for (i = 100; i < conv.length; i++) {

        conv1[i]=0;

}

conv1 = Array.deleteValue(conv1,0);              //New Convolution array after cutting and out of zero values


//Finding local minima

MINIMA=Array.findMinima(conv1, 10);

MINIMA = Array.deleteValue(MINIMA,0);

if (MINIMA.length>0) {

MINIMA_POS=Array.rankPositions(MINIMA);}

else{

MINIMA=newArray(2);

MINIMA_POS=newArray(1);

MINIMA_POS[0]=1;}

Min_pos=MINIMA[MINIMA_POS[0]];//Saving position of absolute min


//Find absolute max in convoluted function

Array.getStatistics(conv1, min1, max1, mean, stdDev);

for (i = 0; i < conv1.length; i++) {
```

```
            if(conv1[i]==max1){

                        Max_pos=i; //Saving the position of aboslute max

                        }

            }


MAXIMA=Array.findMaxima(conv1, 10);  //Maxima array

MAXIMA_POS=Array.rankPositions(MAXIMA);

Array.getStatistics(conv1, min2, max2, mean, stdDev); //Min2 and Max2 are the absolute min and max of convoluted function


//Special cases managment

if(MAXIMA.length==1){ //Case 1: No maxima found, so image is closed (No SST in the image or gray-scale settings are wrong)

            saveAs("png", dir2+name+".tif");

            close();

   close();

   con=1;}else{

//Analysing max position

for (i = 0; i < conv1.length; i++) {

            if(conv1[i]==max2){

            h=i;

            }

            }

diff=pos1-h; //Max position


//Following cases are describing a different position between abosolute max in the original histogram and convoluted one (Convlution changed the absolute max position)

//Case 2: Convoluted max is on the left of absolute max

if(diff>MAXIMA[MAXIMA_POS[1]]){

            Max_pos=MAXIMA[MAXIMA_POS[1]];

            }
```

```
//Case 3:Convoluted max is on the righ of absolute max
if(diff<0){
Max_pos=MAXIMA[MAXIMA_POS[0]];
            }

//Plot creates (If is necessary to visual the histogram with convolution)
//xValues=Array.getSequence(histogram.length);
/*Plot.create("Conv "+deg-1, "Num", "Count", xValues,histogram);
Plot.add("line", xValues, conv1);
Plot.show();*/

//Convolution implies a shift of the original function and it is necessary to calculate, because the ideal threshold value must be chosen on the original Histogram (not convoluted)

//Shift calculation
risc=pos1-Max_pos;
min_fin=Min_pos+risc;

/*______________________________________________________________________________*/
//End of convolution section: RETURN=min_fin (Ideal threshold min)

/*______________________________________________________________________________*/
//Automatic threshold step
            selectWindow(name1);
   setThreshold(min_fin, 255);
   setOption("BlackBackground", true);
   setForegroundColor(255, 255, 255);
   setBackgroundColor(0, 0, 0);
   run("Convert to Mask");
   run("Invert");
   run("Set Scale...", "distance=1 known=1 pixel=1 unit=um");
```

```
//Correction tools
   run("Close-");
   run("Fill Holes");
   run("Analyze Particles...", "size="+low_area_lim+"-Infinity pixel show=Masks include"); //Allows to exclude other particles except SST
   run("Clear Results");
   run("Wand Tool...", "tolerance=20 mode=Legacy");
   getDimensions(width1, height1, channels, slices, frames);

//Finding Edges ot SST
   run("Invert");
   run("Find Edges");
   run("Invert");

//Iverting color of Foreground and Background
        setForegroundColor(0, 0, 0);
        setBackgroundColor(255, 255, 255);

//In order to focus wand only on SST edges it is used "Wand tool" on the background of Image, above and below SST, to make sort of "negative" of it

//Obtaing upper wand points (in order to be sure that wand select the right portion of image)
        makeRectangle(0, 0, width1, 20);
        run("Fill");
        doWand(1, 1);
        roiManager("add");

//Obtaing lower wand points
        makeRectangle(0, height1, width1,20);
        run("Fill");
        doWand(1, height1-1);
        roiManager("add");
```

```
//Combining ROIs
    roiManager("Select", newArray(0,1));
    roiManager("Combine");
    roiManager("Add");
    roiManager("Select", newArray(0,1));
    roiManager("Delete");
    newImage("Untitled", "8-bit black", width1, height1, 1);
    roiManager("Select", 0);
    run("Invert");
    roiManager("Show All");
    roiManager("Show None");
    run("Invert");
    roiManager("Select", 0);
    roiManager("Delete");
    roiManager("Show All");
    roiManager("Show None");
    run("Set Measurements...", "area centroid fit display redirect=None decimal=2");
    run("Analyze Particles...", "size="+low_area_lim+"-Infinity show=Masks include display add");

//ROIs control
ROI_count=roiManager("count"); //Saving ROIs number (must be =1)
fl=0;
if(ROI_count==1){
run("Clear Results");
getDimensions(width2, height2, channels, slices, frames);
makeRectangle(0, 414, width2, 90);
run("Measure");
roiManager("Select", 0);
run("To Bounding Box");
roiManager("Add");
roiManager("Select", 1);
```

```
roiManager("Measure");

roiManager("Select", 1);

roiManager("delete");

rect_axes=getResult("Major", 0);

roi_axes=getResult("Major", 1);

if(rect_axes>roi_axes){fl=1;}


//Saving ROIs number (must be =1)

}}} while (param==0) //End of Do command


if(con==0){

if(ROI_count>1 || fl==1 || ROI_count==0){ //Excluding unwanted cases: ROIs count more than 1 (discontinuous ROI), ROIs count 0 (No ROIs found) or Uncomplete ROI (fl=1)

saveAs("png", dir2+name+".tif"); //Saves discarded images as mask in the corresponding folder


if(ROI_count==0){    //Clearing ROI Manager

}else{roiManager("Delete");}


close(); //Closing all images open

close();

close();

close();

    }else{ //Right case: The ROI must be saved


        //Correcting edge: Excludes a small egeds portion of ROI which is not significative

        width = w;

        height = h;

        makeRectangle(0,0,xth,h);

        roiManager("Add");

        makeRectangle(w-xth,0,xth,h);

        roiManager("Add");

        x= newArray(1,2);
```

```
            roiManager("Select", x);
            roiManager("Combine");
            run("Make Inverse");
            roiManager("Add");
            x= newArray(0,3);
            roiManager("Select", x);
            roiManager("AND");
            roiManager("Add");
            x= newArray(0,1,2,3);
            roiManager("Select", x);
            roiManager("Delete");
            roiManager("Select", 0);
            saveAs("Selection", dir3+fname+".roi");
            roiManager("Select", 0);
            roiManager("Delete");
            getSelectionCoordinates(a,b);

//Closing all images open
close();
close();
close();
close();
close();
}
}
}
}
/*___________________________________________________________________________________*/
//End of Processing image function

//Clearing Result Window
selectWindow("Results");
```

```
run("Close");


//Printng time of calulation and message of ending Analysis
if(lag==1){
	TIME2=getTime();
	sec=round((TIME2-TIME1)/1000);
	min=floor(sec/60);
	if(min>=1){
	sec1=sec-min*60;
	print("Analisi Ultimata, creata cartella con le ROIs\nTime of processing: "+min+" min "+sec1+"
sec");
	}
	}else{
	print("Analisi Ultimata, creata cartella con le ROIs\nTime of processing: "+sec+" sec");}
```
