# Supplementary material for: An Automatic on Top Analysis of Single Scan Tracks to Evaluate the Laser Powder Bed Fusion Building Parameters
Source: Materials (Basel). 2021 Sep 9;14(18):5171. doi: 10.3390/ma14185171 (PMC8472349; doi:10.3390/ma14185171)
Supplement: Supplementary file 1 [file materials-14-05171-s001.zip › Script 2.pdf]

```
//Script 2: Analyzing ROIs applying regularity indexes

//INPUT: "ROI" Folder with files .roi, obtained by Script 1

//OUTPUT: it is a .CSV file with the value of each regularity index for each ROI analyzed

//Measure setting and batch mode
extension = ".roi"; //File extension definition
row1=0;            //Result table first row
rotation_mode = true; //Opening rotation mode

lag=0;                                                          //Ending Analysis
Condition

run("Set Measurements...", "area fit display redirect=None decimal=2"); //Measurment settings

run("Clear Results");                                           //Clear "Results" table, in order to have
starting condition
setBatchMode(true);                                             //Starting Batch Mode

//Input/output Folders management
dir1 = getDirectory("Choose Source Directory ");//User choose the starting folder (Images folder)
dir2= dir1 + "Results\\";                  //Results Folder
File.makeDirectory(dir2);                  //Folders Creation
table1="Res";                          //Results table creation
Table.create(table1);
TIME1=getTime();                          //Time variable of starting analysis (Used for TOC)

//Reading files in input Folder
```

```
list = getFileList(dir1);                    //Saves Images Labels in "list" Array

for (i=0; i<list.length; i++) {

if (endsWith(list[i], "/"))                  //End analysis condition: If a generic "list" is not an image, is saved with
"/" at the end. When the folder is out of images to read, analysis stops.

            lag=1;                                                                  //Updates exit flag

        else if (endsWith(list[i], extension))

          processImage(dir1, list[i]);       //Applies "Processing image funcion" to each image

}


/*______________________________________________________________________________*/

//Processing image function

function processImage(dir1, name) {


open(dir1+name);                                                                         //Opening the image to
process


// Extracting Image name and cut the file extension from it

dotIndex = indexOf(name, ".");

fname = substring(name, 0, dotIndex);


setBatchMode(false);                                              //Allows to see the process on going, if is
set "True" the script will work in background


//Saving image dimension

getDimensions(width, height, channels, slices, frames);


//Setting measurments and getting ROIs properties

run("Set Measurements...", "centroid perimeter fit display redirect=None decimal=2");

roiManager("add");                        //Adding ROI to roi manager

roiManager("Select", 0);

run("Measure");

angle = getResult("Angle");               //Getting ROI angle

x=getResult("X");                                                       //Getting ROI position
```

```
y=getResult("Y");
run("Clear Results");

//ROI Rotation Step
if (rotation_mode==true){
        roiManager("Select", 0);
        if (angle>90){                //Getting right rotation angle
            angle = 180-angle;
            angle = 0-angle;
        }
        run("Rotate...", "rotate angle="+angle);
        roiManager("Add");            //Adding rotate ROI
        roiManager("Select", 0);      //Deleting previous ROI (not rotate one)
        roiManager("Delete");}

//Correcting selection step: in order to applying regularity indeces to ROIs it is necessary to fill ROI (in black or white, depending on background) and to reselect it
roiManager("Select", 0);
setForegroundColor(255, 255, 255);
run("Fill", "slice");
roiManager("Select", 0);
roiManager("Delete");
doWand(x, y);
roiManager("add");

//Regularity indeces Application (Indeces are Functions applied on main code)

PERIM_Ireg1=Perim_Ireg1();                    //Applying Index 1

RZ=Rz();                                      //Applying Index 2

MEAN_STD=width_STD_Ireg2();        //Applying Index 3
```

```
//Setting table with results
selectWindow("Res");
Table.set("Label", row1, fname);                              //ROI label

//Setting Index 1 (Perimeter) with board measures and final perimeter
Table.set("Perim.", row1, PERIM_Ireg1[0]);
Table.set("Board lf", row1, PERIM_Ireg1[1]);
Table.set("Board rg", row1, PERIM_Ireg1[2]);
Table.set("Perim_final",row1, PERIM_Ireg1[3]);

//Setting Index 2
Table.set("Rz",row1,RZ);

//Setting Index 3 and ROI width
Table.set("Mean_width", row1, MEAN_STD[0]);
Table.set("StdDev_wid", row1, MEAN_STD[1]);
Table.set("Num_Inter", row1, MEAN_STD[2]);   //Number of intersection/width measures



Table.update;
row1++;              //Updating table row
run("Clear Results");         //Clearing results and ROI manager
roiManager("delete");
close();             //Closing current image
}
Table.save(dir2 + "results.csv"); //Table saving as a ".CSV" file
selectWindow("Res");          //Closing all windows opened
run("Close");
selectWindow("Results");
```

```
run("Close");

//Printing time of calulation and message of ending Analysis
if(lag==1){
	TIME2=getTime();
	sec=round((TIME2-TIME1)/1000);
	min=floor(sec/60);
	if(min>=1){
	sec1=sec-min*60;
	print("Analisi Ultimata, ora creo i file risultati\nTime of processing: "+min+" min "+sec1+" sec");
	}else{
	print("Analisi Ultimata, ora creo i file risultati\nTime of processing: "+sec+" sec");}
}

/*__________________________________________________________________________________*/

//Index N°1: Perimeter

function Perim_Ireg1 (){

ROI_length=2008;                                                    //ROI length choice
roiManager("Select", 0);
run("Interpolate", "interval=1");          //Choosing interpolation
run("Measure");                                                           //Getting
Perimeter
Perim=getResult("Perim.", 0);

Roi.getCoordinates(xpoints, ypoints);        //Getting ROIs coordinates
r=xpoints.length;

//Edges counters
zeros=0;
```

```
ex=0;

//Edges arrays
ylf=newArray(xpoints.length);
yrg=newArray(xpoints.length);
Array.getStatistics(xpoints, min0, max0, mean, stdDev);//Getting Max and Min of ROI in order to save Edges

//Approximating edges length and saving  (It is considered and approximation because edges length is calculated as difference between Max and Min values, of corresponding edge Y coordinates)
j=0;
h=0;
for (i = 0; i < xpoints.length; i++){
	if(xpoints[i] <= (min0+5) ){ //Left edge
	  ylf[j]=ypoints[i];
	  j++;
	  zeros++;
		}
	if(xpoints[i] >= (max0-5)){ //Rigth edge
		yrg[h]=ypoints[i];
		h++;
		ex++;
		}

	}

// Deleting 0 values from edge Arrays
ylf=Array.deleteValue(ylf, 0);
yrg=Array.deleteValue(yrg, 0);

//Getting Max and Min from edges Arrays for length calculation
Array.getStatistics(ylf, min1, max1, mean, stdDev);
```

```
Array.getStatistics(yrg, min2, max2, mean, stdDev);

//Length edges calculation
dlf=max1-min1;
drg=max2-min2;

//Final perimeter calculation (Perimeter without edges)
Perim_fin=Perim-dlf-drg;
Perim_fin=Perim_fin-2*ROI_length;	//Deducting the double length of the ROI to the Perim_Final

a=newArray(Perim, dlf, drg, Perim_fin); //Returnig an array with Initial Perimeter, Left Edge length, Right Edge Length and Final Perimeter
return a;
}

/*____________________________________________________________________________________*/

//Index N° 2: Rz

function Rz(){
roiManager("Select", 0);
getSelectionCoordinates(XR,YR);				//Getting and Saving ROI coordinates
l = (XR.length);
x = newArray(l);
y = newArray(l);
x=XR;
y=YR;

Array.getStatistics(y, min_y, max_y, mean_y, dst_y); //Getting y coordinates statistics in order to split upper contour and lower contour
l = x.length;
sup = newArray(l);			//Arrays definition for upper and lower contour coordinates
```

```
x_sup = newArray(l);
inf = newArray(l);
x_inf = newArray(l);

//Counters
j = 0;
k = 0;

//Contours splitting and saving in correspondig arrays (Discarding 20 pixel from both sides)
for (i=0; i<l; i++){
	if (y[i]<mean_y){
				sup[j] = y[i];
				x_sup[j] = x[i];
				j++;
			}
			   if (y[i]>mean_y){
				inf[k] = y[i];
				x_inf[k] = x[i];
				k++;
			}

	  }

//Deleting 0 values in new arrays
y_sup = Array.deleteValue(sup,0);
x_sup = Array.deleteValue(x_sup,0);
y_inf = Array.deleteValue(inf,0);
x_inf = Array.deleteValue(x_inf,0);

y_sup=Array.slice(y_sup,15,y_sup.length-15); //Trimming new arrays
y_inf=Array.slice(y_inf,15,y_inf.length-15);
```

```
Array.getStatistics(y_sup, min_ys, max_ys, mean_ys, dst_ys); //Get the statistic of arrays for further calculations

Array.getStatistics(y_inf, min_yi, max_yi, mean_yi, dst_yi);


//Maxima and Minima calculation in single slices

divisions=5;                    //Number of slices chosen


Seq=Array.getSequence(divisions+1);        //Slices division for upper and lower contour

Seq=Array.deleteValue(Seq, 0);

Slices_sup=y_sup.length/divisions;

Slices_inf=y_inf.length/divisions;

Array.getStatistics(x_sup, min, max, mean, stdDev);


//Array defition to save the corresponding information about Peaks and Valley

New_sup=newArray(y_sup.length);

New_inf=newArray(y_inf.length);

Slices_sup=floor(Slices_sup);

Slices_inf=floor(Slices_inf);

VALLEYS_inf=newArray(divisions);

PEAKS_inf=newArray(divisions);

VALLEYS_sup=newArray(divisions);

PEAKS_sup=newArray(divisions);


//Counters

h=0;

i=1;


while(i<y_inf.length){                                  //Lower contour cycle where are saved the highest peak and lowest valley for each ROI slice

New_inf[i]=y_inf[i];

cont=i/Slices_inf;

for (j = 0; j < Seq.length; j++) {
```

```
if(cont==Seq[j]){

run("Add Selection...");

New_inf_2=Array.copy(New_inf);                                    //Updating corresponding slice array

New_inf_2=Array.deleteValue(New_inf_2, 0);

Array.getStatistics(New_inf_2, min, max, mean, stdDev);                  //Saving max and min (peak and
valley) of current slice

VALLEYS_inf[h]=min;                              //Updating valleys and peaks arrays

PEAKS_inf[h]=max;

h++;

New_inf=Array.fill(New_inf,0);

i=i+50;                                                           //Correction in order to exclude peaks on
the edge of the single slice

}


if(i==y_inf.length-1 && h<VALLEYS_inf.length){                           //Last slice manager

New_inf_2=Array.copy(New_inf);

New_inf_2=Array.deleteValue(New_inf_2, 0);

Array.getStatistics(New_inf_2, min, max, mean, stdDev);                  //Saving max and min (peak and
valley) of current slice

VALLEYS_inf[h]=min;
        //Updating valleys and peaks arrays

PEAKS_inf[h]=max;

}

}

i++;

}


//Reinitializating counters

h=0;

i=1;


while(i<y_sup.length){                                                                      //Higher
contour cycle where are saved the highest peak and lowest valley for each ROI slice
```

```
New_sup[i]=y_sup[i];
cont=i/Slices_sup;
for (j = 0; j < Seq.length; j++) {
if(cont==Seq[j]){
run("Add Selection...");
New_sup_2=Array.copy(New_sup);                                    //Updating corresponding slice array
New_sup_2=Array.deleteValue(New_sup_2, 0);
Array.getStatistics(New_sup_2, min, max, mean, stdDev);          //Saving max and min (peak and valley) of current slice
VALLEYS_sup[h]=min;
        //Updating valleys and peaks arrays
PEAKS_sup[h]=max;
h++;
New_sup=Array.fill(New_sup,0);
i=i+50;
        //Correction in order to exclude peaks on the edge of the single slice
}
if(i==y_sup.length-1){                                            //Last slice manager
New_sup_2=Array.copy(New_sup);
New_sup_2=Array.deleteValue(New_sup_2, 0);
Array.getStatistics(New_sup_2, min, max, mean, stdDev);          //Saving max and min (peak and valley) of current slice
VALLEYS_sup[h]=min;
        //Updating valleys and peaks arrays
PEAKS_sup[h]=max;
}
}


i++;
}


//Cylce for calculation of mean highest peak and lowest valley (Applying Rz classic formula)
```

```
for (i = 0; i < VALLEYS_sup.length; i++) {
Sum_peak_sup=Sum_peak_sup+PEAKS_sup[i];
Sum_val_sup=Sum_val_sup+VALLEYS_sup[i];
Sum_peak_inf=Sum_peak_inf+PEAKS_inf[i];
Sum_val_inf=Sum_val_inf+VALLEYS_inf[i];
}
Mean_sup=(Sum_peak_sup-Sum_val_sup)/5;
Mean_inf=(Sum_peak_inf-Sum_val_inf)/5;

res=(Mean_sup+Mean_inf)/2; //Returning the mean value of Rz between upper and lower contour
return res;
}

/*____________________________________________________________________________*/

//Index N°3: Mean width calculation and Standard deviation
function width_STD_Ireg2 (){
//User choice
line_num=10;                    //User can choose the number of intersections for the measure



line_num=line_num+1;                                            //The number chosen is higher
than real intersection number of 1 unit, because the measure exclude ROI edges
amp=newArray(line_num-1);            //Width array
for (i = 0; i <line_num-1; i++) {
        width_Est=width/line_num;           //Roi Division
        makeLine(width_Est*(i+1),0, width_Est*(i+1),height);  //Line Making to calculate width
        roiManager("Add");

 //ROIs intersection (Script from Imagej site): intersection between line maked and pre-existent ROI
 setBatchMode(true);
 roiManager("Select", 0);
```

```
  orig = getImageID();
  run("Create Mask");
  rename("Mask1");
  mask1 = getImageID();
  roiManager("Select", 1);
  run("Create Mask");
  mask2 = getImageID();
  imageCalculator("AND", mask2, mask1);
  run("Create Selection");
  roiManager("Add");
  selectImage(orig);
  roiManager("Select", 2);


  //Width Calculation
  run("Set Measurements...", "bounding redirect=None decimal=2");
  run("Clear Results");
  roiManager("Select", 2);
  run("Measure");
  amp[i]=getResult("Height");                                        //Updating Width
array with calculated value
  roiManager("Select", 1);
  roiManager("Delete");
  roiManager("Select", 1);
  roiManager("Delete");
  setBatchMode(false);


}
//End of cycle: now "Amp" array is filled with all width calculated from each ROI slice


Array.getStatistics(amp, min, max, mean_wid, stdDev_wid);      //Getting "Amp" statistics: mean and
STD_DEV of all his values, so ROI widths
b=newArray(mean_wid, stdDev_wid, line_num-1);            //Returnig an array with: Mean Width, STD_DEV
and number of intersection chosen
```

```
return b;

}
```
